# Polypyrrole-multi walled carbon nanotube hybrid material supported Pt NPs for hydrogen evolution from the hydrolysis of MeAB at mild conditions

**DOI:** 10.1038/s41598-019-55030-z

**Published:** 2019-12-06

**Authors:** Yasar Karatas, Esra Kuyuldar, Hilal Acidereli, Mehmet Gulcan, Fatih Sen

**Affiliations:** 1 Chemistry Department, Faculty of Science, Van Yüzüncü Yıl University, Zeve Campus, 65080 Van, Turkey; 2 Sen Research Group, Biochemistry Department, Faculty of Arts and Science, Dumlupınar University, Evliya Çelebi Campus, 43100 Kütahya, Turkey

**Keywords:** Catalyst synthesis, Heterogeneous catalysis

## Abstract

Herein, we report a facile method for the preparation of polypyrrole-multi walled carbon nanotube hybrid material including Pt nanoparticles (Pt@PPy-MWCNT NPs) and the use in methylamine borane (MeAB) for hydrolysis reaction at mild conditions. The prepared catalyst of Pt@PPy-MWCNT NPs was characterized by some advanced analytical methods. The catalytic experiments showed the Pt@PPy-MWCNT NPs can catalyze MeAB in aquatic solution with high catalytical performance at mild conditions. The reaction rate of catalytic hydrolysis with Pt@PPy-MWCNT NPs was found to be -d[CH_3_NH_2_BH_3_]/dt = + d[H_2_]/3dt = k_obs_[Pt@PPy-MWCNT]^1.19^ [MeAB]^0.88^. The TOF value for the hydrolysis of MeAB catalyzed with Pt@PPy-MWCNT NPs was detected to be 10234.2 1/h (170.57 1/min) which is very high compared with TOF values found for other catalysts. Enthalpy, entropy and activation energy for the hydrolysis of MeAB were calculated to be 31.57 kJ mol^−1^, −119.97 J mol^−1^ K and 34.27 kJ mol^−1^, respectively.

## Introduction

Hydrogen (H_2_) is a fuel known all over the world as a fuel. The introduction of hydrogen in transportable electronic tools or carriers, it is required to use an H_2_ tank or “on board” in the production. Many studies have been reported about H_2_ storage methods^1,2^, obtaining H_2_ from hydrocarbons by the onboard method^3,4^. Furthermore, it is not possible to use all the methods due to the low volume and gravimetric efficiency of the storage of hydrogen, the high-temperature processability of hydrocarbons and the safety difficulties. In order to use H_2_ as fuel, it is necessary to make efficient storage of H_2_ with a reliable method. In recent studies, various different materials like sorbents^5^, metal hydrides^6^, and chemical hydrides process^7–10^ have been applied in the developing hydrogen storage. Among these materials, amine-boranes have taken great deal attention because their hydrogen contents are high and hydrogen release is applicable^11^. Recently, as an amine-borane compound, ammonia-borane (NH_3_-BH_3_, AB) has been noted as a high-potential source for hydrogen storage due to its hydrogen content of 19.6% wt., high durability, and environmentally friendly properties^12,13^. For this purpose, solid-phase thermolysis^14^, catalytic hydrogen evolution in anhydrous solvents^15^, and hydrolysis^16,17^ can be used to provide the release of H_2_ from amine-borane compounds. The hydrolysis of the AB in the presence of the suitable catalyst may yield 3 moles of hydrogen per 1 mol of AB (Eq. (1)). This is one of the most appropriate methods of portable hydrogen storage^18^. Besides, methylamine borane (CH_3_NH_2_-BH_3_, MeAB), an amine borane derivative, has a hydrogen content of 11.1% by weight. Moreover, as given Eq. (2) H_2_ gas can take place with % 100 yields. Although there are many studies related to the AB and its catalytic mechanism, the catalytic dehydrogenation mechanism of MeAB has remained unexplored^19,20^.

1 $${{\rm{NH}}}_{3}{{\rm{BH}}}_{3}+2\,{{\rm{H}}}_{2}{\rm{O}}\,\underset{{\rm{RT}}}{\overset{{\rm{catalyst}}}{\longrightarrow }}\,{{\rm{NH}}}_{4}{{\rm{BO}}}_{2}+3{{\rm{H}}}_{2}$$

2 $${{\rm{CH}}}_{3}{{\rm{NH}}}_{2}-{{\rm{BH}}}_{3}+2\,{{\rm{H}}}_{2}{\rm{O}}\,\underset{{\rm{RT}}}{\overset{{\rm{catalyst}}}{\longrightarrow }}\,{{\rm{CH}}}_{3}({{\rm{NH}}}_{3}){{\rm{BO}}}_{2}+3\,{{\rm{H}}}_{2}$$

The Lewis acid, which is formed by taking the electron from the Lewis base, has much better controlled in the reduction rate, and selective reduction of the metal ions on conventional reducing chemicals such as in borohydrides^21^. The alkyl substitution at nitrogen is increased while the strength decreases as the following order: Me_3_N-BH_3_ <Me_2_NH-BH_3_ <MeNH_2_-BH_3_ <BH_3_. In literature, there are limited studies on developing reducing chemicals for the metal-based nanoparticles synthesis^22^. Thus, the studies are carried out to develop low-cost, high-efficiency and stable catalysts for hydrogen production. Although there are many studies related to the development of novel catalysts, only a few studies are given associated with hybrid materials and their usage as catalyst systems^14–22^. For this reason, in this study, a polypyrrole-multi walled carbon nanotube hybrid material supported Pt nanoparticle (Pt@PPy-MWCNT NPs) was used for hydrogen evaluation from the hydrolysis of MeAB and complete conversion of MeAB to products as seen in equation 2 was achieved. Using trace of concentration of Pt@PPy-MWCNT catalyst in an aquatic mixture of MeAB at room temperature, 3 mmol H_2_ release from 1 mmol MeAB was achieved. Additionally, the kinetic studies of MeAB catalyzed Pt@PPy-MWCNT NPs were also performed and activation parameters including entropy (ΔS^#^), enthalpy (ΔH^#^), activation energy (Ea) were calculated.

## Results and Discussion

### Structural and morphological features of Pt@PPy-MWCNT NPs

**Figure 1:**
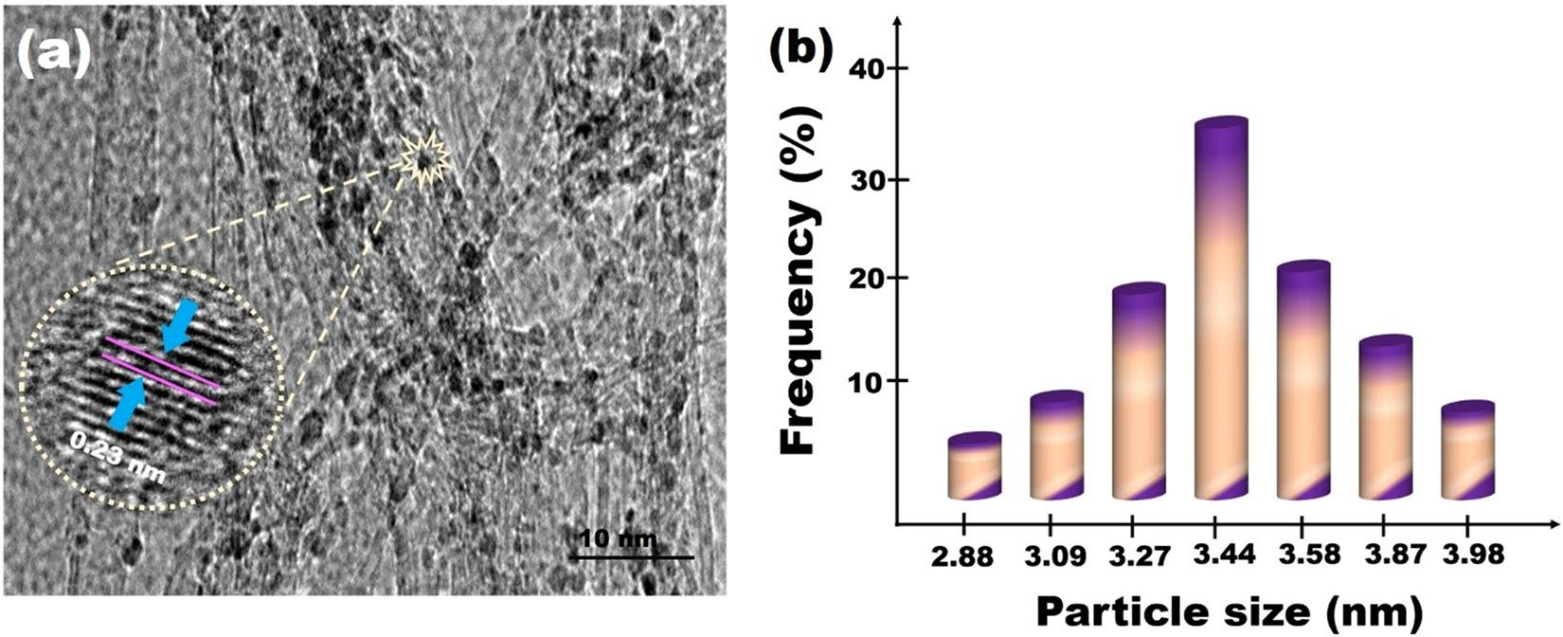
(**a**) TEM image and HR-TEM image and (**b**) particle size histogram of Pt@PPy-MWCNT NPs.

Some sophisticated techniques, including TEM, XRD, Raman spectroscopy etc have been used to study the surface and structural composition of PPy-MWCNT and Pt@PPy-MWCNT NPs. Detail characterization and explanations of PPy-MWCNT have attached to the supporting information and shown in Figs. S1–S3. TEM and HR-TEM images of the Pt@PPy-MWCNT NPs are given in Fig. 1a and S4. The particle size histogram for Pt@PPy-MWCNT NPs was shown in Fig. 1b. TEM analysis detected a goog Platinium dispersion on the support and a spherical Pt metals shape having a mean particle-sized ranging from 3.44 to 4.07 nm that show few agglomerations have occurred on the surface of PPy-MWCNT supports (Fig. 1a,b). The enlarged image indicates that the surface consists of a coating of platinum nanoparticles with inter-fringe distances of 0.23 nm, equal to the fcc platinum Pt (111) plane^21^.

**Figure 2:**
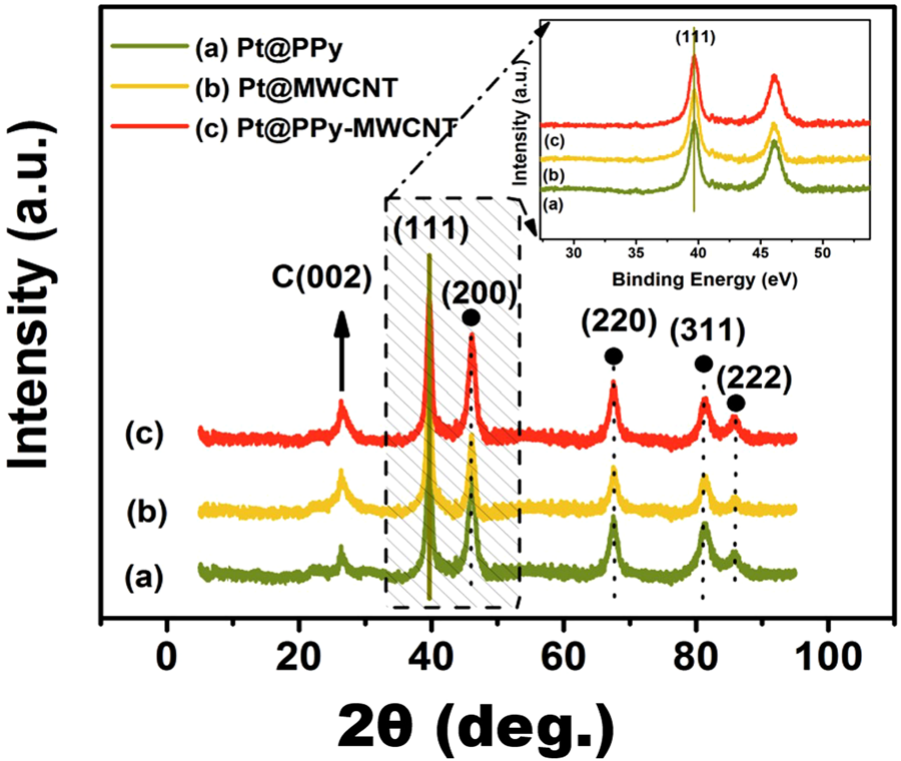
XRD analyses of (**a**) Pt@Ppy, (**b**) Pt@MWCNT and (**c**) Pt@PPy-MWCNT NPs.

XRD studies were carried out to detect the crystalline formation of Pt@PPy-MWCNT NPs (containing 2.78 ± 0.02 wt % Pt). XRD analysis, given in Fig. 2, indicated no composition change in PPy-MWCNT because of decorated with reduced platinum metals. It can be said that Pt@PPy-MWCNT NPs maintained their platin metals and PPy-MWCNT support material based composition after preparation study. Briefly, XRD studies revealed that there was no change of chemical composition in the form of PPy-MWCNT that make Pt@PPy-MWCNT an ideal nanoparticle. On the other hand, diffraction peaks of Pt@PPy-MWCNT NPs in the XRD pattern (Fig. 2(c)) at 2θ = 39.9°, 46.3°, 67.5°, 81.6° and 85.5°, compatible with (111), (200), (220), (311) and (222), respectively. These data detect the face-centered cubic form of Pt that ascribed to (JCPDS Card 04-0802) shows Pt crystalline form of Pt^22,23^.

**Figure 3:**
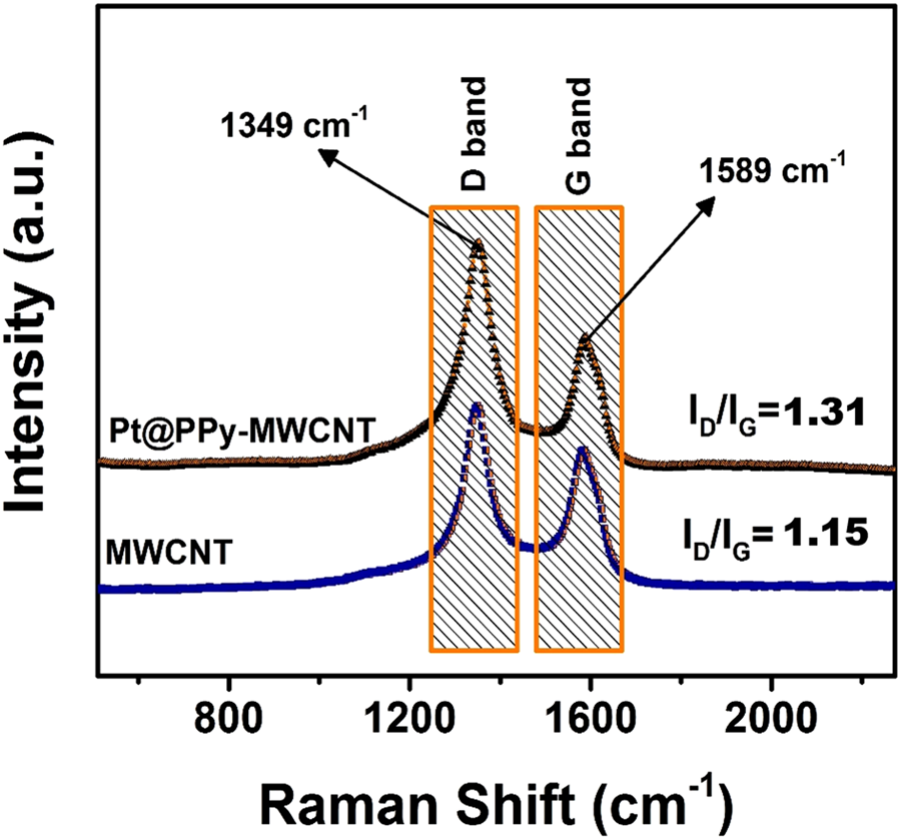
Raman spectra of MWCNT and Pt@PPy-MWCNT NPs.

The representation of the MWCNT and PPy-MWCNT supported platinum nanoparticles by Raman Spectroscopy is shown in Fig. 3. As it can see in Fig. 3, the peaks of carbon products corresponding to the D and G band are observed at 1349 cm^−1^ and 1589 cm^−1^, respectively. The density ratios of D-G bands (I_D_/I_G_) are investigated to identify the rate of graphitization and the error margin of the carbon-containing materials. I_D_/I_G_ ratio is increased from 1.24 to 1.31 which means that the MWCNT was functionalized by PPy and Pt nanoparticles. The functionalization of MWCNT results in increasing of D band peak density.

**Figure 4:**
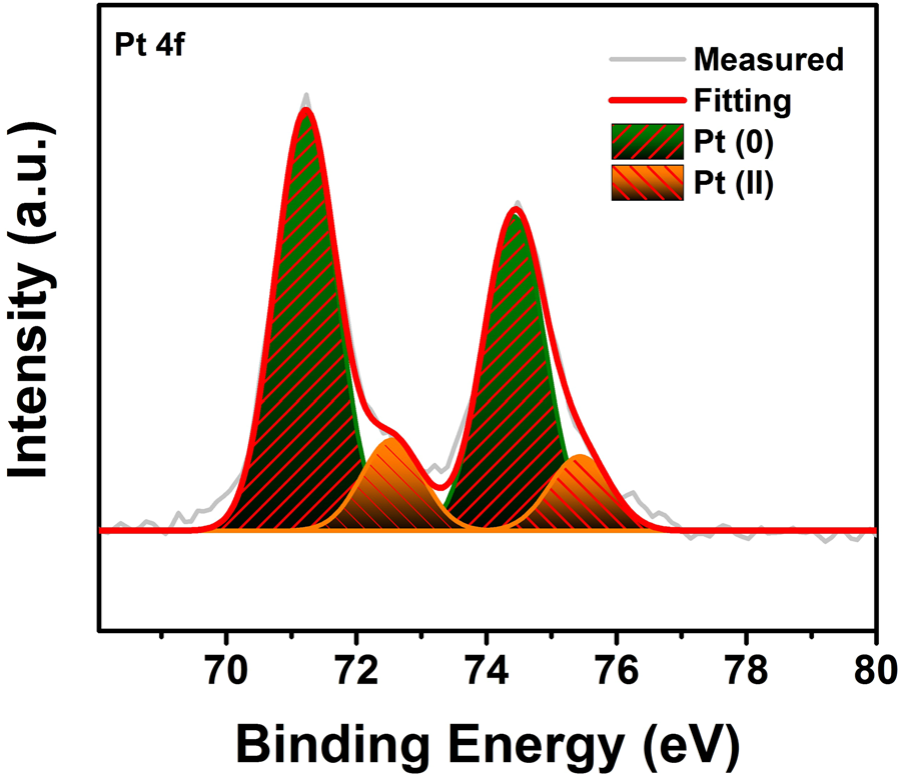
Pt 4 f region of X-ray photoelectron spectrum of Pt@PPy-MWCNT NPs.

The oxidation and electronic state of platin metals present in the composition of the prepared catalyst (Pt@PPy-MWCNT) were examined using X-ray photoelectron spectroscopy (XPS). The XPS results given in Fig. 4 show that the two peaks at the 71.0 and 72.9 eV for the 4 f region are attributed to the metallic Pt (0) and Pt (II) platinum types^22^.

### The hydrolysis of MeAB catalyzed by Pt@PPy-MWCNT NPs

**Figure 5:**
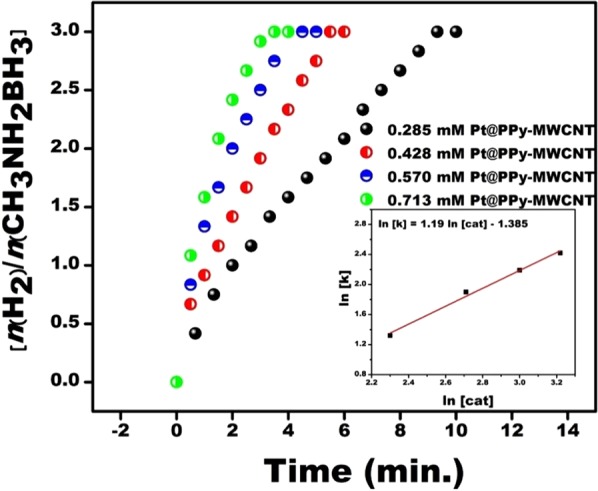
Plot of ration of nH_2_/CH_3_NH_2_BH_3_ versus time for MeAB (50 mM in 5 mL H_2_O) and plot of lnk versus ln[cat.] (inset) with different Pt@Py-MWCNT NPs ([Pt@PPy-MWCNT NPs] = 0.285, 0.428, 0.570 and 0.713 mM) for MeAB hydrolysis at mild conditions.

The catalytical reaction experiments were conducted using a set composed of a flask tube having two necks (Schlenk) connected to a graduated tube for MeAB dehydrogenation catalyzed Pt@PPy-MWCNT NPs. One neck connected to a circulator operating to adjunct temperature and the other connected to the graduated tube to determine the released hydrogen volume. A standard reaction procedure initiated by moving the necessary quantity of catalyst to Schlenk stirred for 15 min at 600 rpm to achieve a temperature of equilibrium. At the end of this time, 11.25 mg of MeAB was applied to the reaction medium, and Schlenk was quickly shut down and the reaction started with a chronometer. The released H_2_ amount was recorded by observing a change of water level in the graduated tube. To detect the effect of catalyst amount to the MeAB catalytical reaction, different catalyst concentrations from 2.85 × 10^−4^ M to 7.13 × 10^−4^ M were tested at room conditions and the finding of these experimental results is given in Fig. 5. The MeAB reaction catalyzed with Pt@PPy-MWCNT NPs initiated very fast and not seen any induction given in Fig. 5 and H_2_ release rated was increased with increasing catalyst concentration. Also, as seen almost the whole hydrogen in the composition of MeAB with the presence of Pt@PPy-MWCNT NPs was released in 2–10 min. The findings of catalyst concentration were evaluated and a linear plot of ln[k] versus ln[cat.] plot was obtained as the seen inlet of Fig. 5 having 1.19 slope. The hydrolysis reaction of MeAB catalyzed by Pt@PPy-MWCNT NPs was the almost first order (1.19^th^-order) based on the concentration of Pt@PPy-MWCNT NPs.

**Figure 6:**
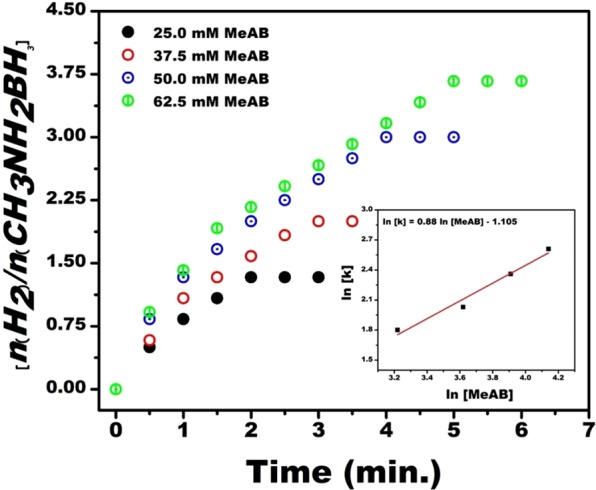
Plot of nH_2_/CH_3_NH_2_BH_3_ versus time for MeAB (50 mM) containing Pt@PPy-MWCNT NPs ([Pt@PPy-MWCNT NPs] = 5.7 × 10^−4^ M in 5 mL H_2_O), and plot of lnk versus ln [MeAB] (inset) for the hydrolysis of MeAB including various MeAB amounts ([MeAB] = 25, 37.5, 50 and 62.5 mM) at mild conditions.

To detect the effect of substrate concentration on the MeAB catalytical reaction, different MeAB concentrations in the range of 25–62,5 mM were tested at room conditions and the finding of these experimental results is seen in Fig. 6. If the graph is plotted for lnk versus ln [MeAB], a linear line will be obtained as shown in Fig. 6 (Inset) and its slope was 0.88. This means the MeAB reaction rate catalyzed by Pt@PPy-MWCNT NPs was the 0.88^th^-order based on the concentration of MeAB. In the light of these results, the equation of the catalytic hydrolysis rate of MeAB including Pt@PPy-MWCNT NPs was attained as follows;

$$-{\rm{d}}[{{\rm{CH}}}_{3}{{\rm{NH}}}_{2}-{{\rm{BH}}}_{3}]/{\rm{dt}}=+\,{\rm{d}}[{{\rm{H}}}_{2}]/3{\rm{dt}}={{\rm{k}}}_{obs}{[\text{Pt}@\text{PPy}-{\rm{MWCNT}}]}^{1.19}{[{\rm{MeAB}}]}^{0.88}$$

**Figure 7:**
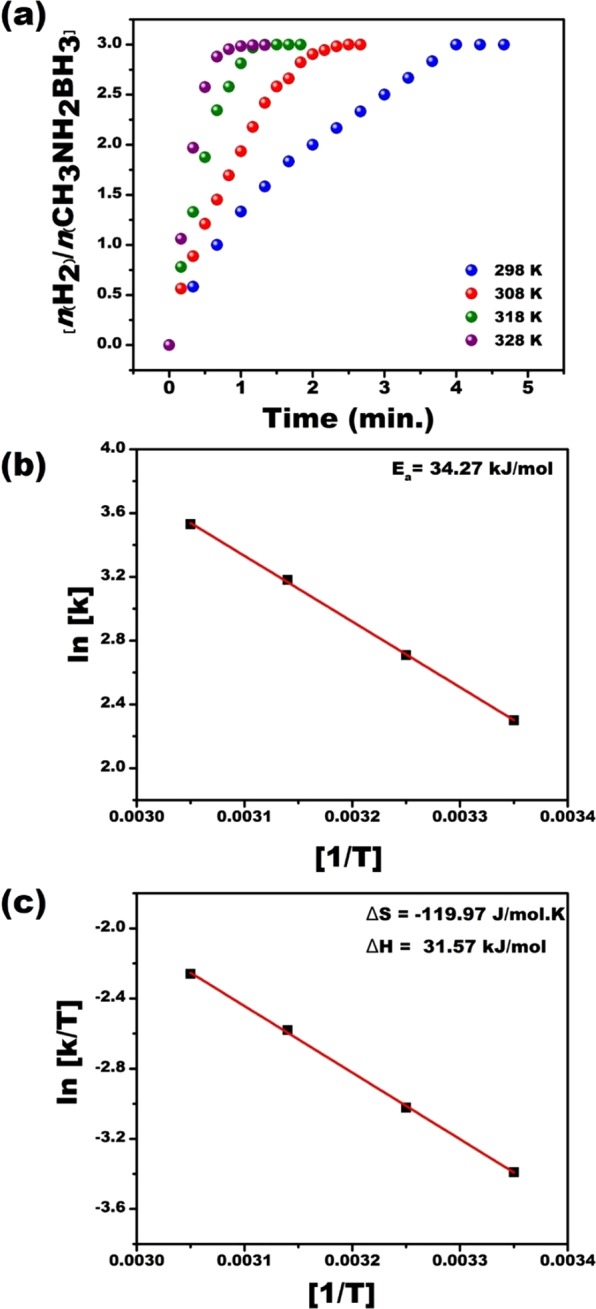
(**a**) Plot of nH_2_/CH_3_NH_2_BH_3_ versus time for MeAB (50 mM in 5 mL H_2_O) beginning with Pt@PPy-MWCNT NPs ([Pt@PPy-MWCNT NPs] = 0.57 mM in 5 mL H_2_O) at varied temperatures within 298–328 K range, (**b**) Arrhenius, and (**c**) Eyring plots of the reactions.

To detect the effect of temperature to the MeAB catalytical reaction, different catalyst amounts in the range of 25–55 °C were tested at room conditions and the finding of these experimental results is seen in Fig. 7. In these experiments, 50 mM CH_3_NH_2_-BH_3_ and 0.57 mM Pt@PPy-MWCNT NPs were used as common parameters. In Fig. 7, the hydrogen release rate was improved by increased temperature. The results obtained from experiments performed at different temperatures were employed to detect activation parameters like ΔS^#^, Ea and ΔH^#^ using Arrhenius and Eyring plots of Fig. 7. These data were found to be ΔS^#^ = −119.97 J mol^−1^ K^−1^, Ea = 34.27 kJ mol^−1^ and ΔH^#^ = 31.57 kJ mol^−1^.

In order to evaluate the catalytical reusable and durable of Pt@PPy-MWCNT, the reuse experiments of catalysts were done and examined as shown in Fig. S5. A typical reuse run was conducted by taking the same amount of catalyst and substrate as tested the other common experiments. The reaction was initiated with this amount of Pt@PPy-MWCNT catalyst and MeAB substrate and waited until the catalytical reaction finished. After the reaction was ended, the same amount of support material taken at the initial was added to the first reaction medium and the reaction was initiated again at room conditions. This procedure was repeated 5 times, the run results are given in Fig. S5, the initial catalytical performance of Pt@PPy-MWCNT catalyst was detected to be 85.4% after the 5^th^ run. The XRD and TEM characterization of Pt@PPy-MWCNT after reuse were shown in Figs. S6 and S7. The results indicate that there is a small amount of agglomeration after reuse as shown in these figures. The initial TOF value for the hydrolysis of MeAB catalyzed with Pt@PPy-MWCNT NPs as a catalyst was detected to be 10234.2 h^−1^ (170.57 min^−1^) that is very high compared with TOF values found for other catalysts as given in Table 1. With this study, we showed the complete conversation MeAB to the products can be achieved using Pt@PPy-MWCNT NPs as a catalyst under mild conditions. We can interpret these results as the synergic effects between supports and metals, high metal contents and ultrafine chemical compositions maybe some of the main reasons for a good catalytical activity. The good stabilization of PPy-MWCNT with platinum metals and enhanced active surface side on the catalysts (according to characterization studies) can be the other reasons in high catalytical performance of Pt@PPy-MWCNT catalyst.

**Table 1:** Comparisons of catalytic activities of the catalysts used for the hydrolysis of MeAB.

Catalyst	TOF*	Ea**	Ref.
Cu_12.6_@Fe_9.8_Co_38.8_Ni_38.8_/graphene	ND	39.69	^25^
Cu_0.2_@Co_0.8_/rGO	ND	55.9	^26^
Rh/graphene	146	16.4	^27^
Co_0.9_Ni_0.1_/graphene NPs	ND	26.78	^28^
Ru_1_@Ni_7.5_/graphene NPs	231	37.01	^29^
Ru/MCM-41	47.60	ND	^30^
Cu_0.1_@Co_0.45_Ni_0.45_/graphene NPs	ND	50.75	^31^
Ag@CoNiFe/graphene	ND	33.53	^32^
Rh_1_Ni_7.5_/graphene	ND	31.26	^33^
Ru@Co/graphene NPs	226	ND	^34^
Cu/*nano*-MIL-101	4.28	34.1	^35^
Rh@PVP40	185.91	43.88	^24^
Ru-Rh@PVP	206.20	43.5	^36^
Pt@PPy-MWCNT NPs	**170.57**	**34.27**	**This study**

*Turn-over frequency (mol H_2_ mol catalyst^−1^min^−1^), **Activation energy (kJ mol^−1^), ND (Not Demonstrated).

## Experimental

### Preparation of methylamine-borane (CH_3_NH_2_BH_3_, MeAB)

MeAB was used for kinetic studies as substrate^24^. For this aim, 0.1 moles of (3.88 g) sodium borohydride (NaBH_4_) and 200 mL anhydrous tetrahydrofuran (THF) were placed in a 250 mL of volume two-neck flask. The resulting mixture was stirred for 30 min at 298 K, 0.1 mol (6.752 g) of methylamine hydrochloride (CH_3_NH_2_∙HCl) was transferred in it (Eq. (3)). The reaction maintained for 24 hours in the N_2_ atmosphere at 298 K. 24 hours later, and the solid product was separated. The residual liquid phase was evaporated to obtain THF. A 100 mL dry sample of ether was transferred to the latest solution and stirred at 0 °C for 2 h, and the solid was filtered. The obtained liquid ether was left to evaporate about 20 °C. After the complete evaporation of, the white solid crystalline formation was observed.

3 $${{\rm{CH}}}_{3}{{\rm{NH}}}_{2}.{\rm{HCl}}+{{\rm{NaBH}}}_{4}\,\underset{{N}_{2},24\,{\rm{h}}}{\overset{{\rm{anhydrous}}\,{\rm{THF}}}{\longrightarrow }}\,{{\rm{CH}}}_{3}{{\rm{NH}}}_{2}-{{\rm{BH}}}_{3}+{{\rm{H}}}_{2}+{\rm{NaCl}}$$

### Synthesis of polypyrrole-multi walled carbon nanotube (PPy-MWCNT) hybrid support material

The following procedure was followed for the preparation of PPy-MWCNT hybrid support material: 20 mg of polypyrrole was added into 25 mL of water and dissolved in ultrasonication for 1 hour. In another beaker, 20 mg of MWCNT was added into 24 mL of water and dissolved by ultrasonication for 1 hour. The obtained solutions were brought together and kept for 30 minutes until thoroughly mixed. The resulting homogeneous mixture was taken into the Schlenk tube, and the synthesis mechanism was established. The ambient conditions were kept stable with the help of water circulation and N_2_ gas. N_2_ gas was allowed to stir for 1 hour in a trapped Schlenk tube. After this process, the final step of the synthesis was carried out by adding reductant. The solution was allowed to stir under reflux for 12 hours at 100 °C. After 12 hours, the prepared solution was washed with ethanol or water to perform the washing operation by centrifugation. After repeated washing, it was allowed to dry in a petri dish and dried under vacuum.

### Synthesis of polypyrrole-multi walled carbon nanotube hybrid material supported Pt metal nanoparticles (Pt@PPy-MWCNT NPs)

PPy-MWCNT nanocomposites synthesis was performed using *in situ* chemical pyrrole monomer polymerization in an aqueous solution containing dispersed MWCNTs. For this purpose, firstly, 24 ml deionized water and 20 mg MWCNTs previously treated with acids were sonicated for 1 hour. The respective suspension was then added to 25 ml of pyrrole and sonicated for 1 hour. The mass ratio of the pyrrole monomer/MWCNTs was 1. An oxidant, ammonium persulfate (APS) aqueous solution was gradually applied to the mixture. The reaction took almost 30 minutes under magnetic stirring. Finally, methanol was added to be able to finish the pyrrole polymerization reaction. After that, a simple, one-step method was applied for the preparation of platinum nanoparticles supported on PPy-MWCNT. Simply, a solution was prepared by dissolving 0.0252 mmol of K_2_PtCl_6_ and 0.025 mg/mL of PPy-MWCNT support in 5 ml of water. The prepared solution was mixed for 2 h and then a 15 mL of sodium boron hydride was added as a reducing agent (NaBH_4_) was transferred into the latest solution. After reducing Pt (IV) into Pt (0), the resulting solution was filtered, and the solid sample was cleaned by washing using abundant pure water. The catalyst sample was dried in a vacuumed oven at 80 °C. The dried samples were stored for further using of experiments conducted with different temperatures, substrate concentrations and catalyst concentrations.

## Conclusions

In this paper, we showed that Pt@PPy-MWCNT NPs can be prepared by using a simple and facile approach and effectively used in the MeAB hydrolysis reaction at mild conditions. The catalytic experiments showed the Pt@PPy-MWCNT NPs can catalyst MeAB in aquatic solution and with high catalytical performance at mild conditions. The reaction rate of catalytic hydrolysis of MeAB include Pt@PPy-MWCNT NPs was found to be -d[CH_3_NH_2_BH_3_]/dt = +d[H_2_]/3dt = k_*obs*_[Pt@PPy-MWCNT]^1.19^ [MeAB]^0.88^. The TOF value for the hydrolysis of MeAB catalyzed as a catalyst using Pt@PPy-MWCNT NPs was detected to be 10234.2 h^−1^ (170.57 min^−1^) that is very high compared with TOF values found for other catalysts as given in Table 1. The main reason for this high catalytic performance can be the synergistic effect formed between Pt metals and PPy-MWCNT support materials. Due to this superior catalytic performance of Pt@PPy-MWCNT NPs in the hydrolysis of MeAB, the prepared this catalyst can be a good candidate for hydrogen evolution from hydrogen storage materials in the usage of fuel cells. Some parameters of activation such as enthalpy, entropy and activation energy for this important reaction were calculated to be 31.57 kJ mol^−1^, −119.97 J mol^−1^ K and 34.27 kJ mol^−1^, respectively.

## Supplementary information

Polypyrrole-multi walled carbon nanotube hybrid material supported Pt NPs for hydrogen evolution from the hydrolysis of MeAB at mild conditions
