# Supplementary material for: Polypyrrole-multi walled carbon nanotube hybrid material supported Pt NPs for hydrogen evolution from the hydrolysis of MeAB at mild conditions
Source: Sci Rep. 2019 Dec 6;9:18553. doi: 10.1038/s41598-019-55030-z (PMC6898010; doi:10.1038/s41598-019-55030-z)
Supplement: Supplementary file 1 — Polypyrrole-multi walled carbon nanotube hybrid material supported Pt NPs for hydrogen evolution from the hydrolysis of MeAB at mild conditions [file 41598_2019_55030_MOESM1_ESM.docx]

Electronic Supplementary Material

**Polypyrrole-multi walled carbon nanotube hybrid material supported Pt NPs for hydrogen evolution from the hydrolysis of MeAB at mild conditions**

Yasar Karatas^a^, Esra Kuyuldar^b^, Hilal Acidereli^b^, Mehmet Gulcan^a*^, Fatih Sen^b*^

*^a^Chemistry Department, Faculty of Science, Van Yüzüncü Yıl University, Zeve Campus 65080 Van, Turkey;*

*^b^Sen Research Group, Biochemistry Department, Faculty of Arts and Science, Dumlupınar University, Evliya Çelebi Campus, 43100 Kütahya, Turkey*

**Materials and Methods**

# Multiwall carbon nanotube (MWCNT), Pyrrole, Ammonium persulfate (APS), Methanol, Anhydrous Tetrahydrofuran (THF), Sodium borohydride (NaBH_4_), Potassium hexachloroplatinate(IV) (K_2_PtCl_6_) were obtained from Sigma Aldrich. The TEM images have been obtained by a JEOL 200 kV TEM instrument. Powder X-ray Diffraction (P-XRD) analysis was performed using a diffractometer with Ultima + theta + theta high resolution goniometer, Cu Kα radiation (Bruker AXS D8-Advanced, 45 kV, 40 mA, λ= 1.54056Å). In the characterization studies, a solution was prepared as described in the above section 2.2 for examination; this solution was centrifuged at 8000 rpm for 15 minutes. The nanoparticles obtained from the centrifuged mixture are cleaned using purified water and ethanol to remove contaminants and the excess of the nanoparticle of Pt@PPy-MWCNT. The obtained nanoparticles were re-dissolved in 5 mL of pure water. One drop of the resulting mixture was added dropwise onto a copper grid and evaporated to dryness under nitrogen. TEM images were used to calculate the mean particle size of Pt@PPy-MWCNT nanocatalyst, to do this, the particles in the TEM images were calculated by counting them. The absorption experiments of the platinum (0) nanoparticles stabilized with PPy-MWCNT were performed with a double-beam Perkin Elmer Lambda UV-VIS-NIR spectrophotometer. FT-IR analyses were taken by Perkin Elmer Spectrum 2. X-ray Photoelectron Spectroscopy (XPS) analysis was done using physical electronics 5800 spectrometers consisted of the hemispherical analyzer and having monochromatic Al Kα radiation (1486.6 eV, the X-ray tube working at 15 kV, 350 W and pass energy of 23.5 keV). Nuclear magnetic resonance (NMR) analysis was performed by a Bruker Avance DPX 400 MHz spectrometer (400.1 MHz for ^1^H NMR; 100.6 MHz and 128.2 MHz for ^11^B NMR). BF_3_(C_2_H_5_)_2_O and Si (CH_3_)_4_ were taken to use as an internal reference for ^11^B-NMR and ^1^H-NMR chemical shifts.





**Fig. S1.** XRD patterns of MWCNT, PPy and PPy-MWCNT

In Fig. S1, the peak value of MWCNT in a and PPy in b can be seen. In c, when the MWCNT and PPy forms a composite, it is observed that the peak intensity decreases due to the composite formed, and the peak value is 25.58˚ is preserved.


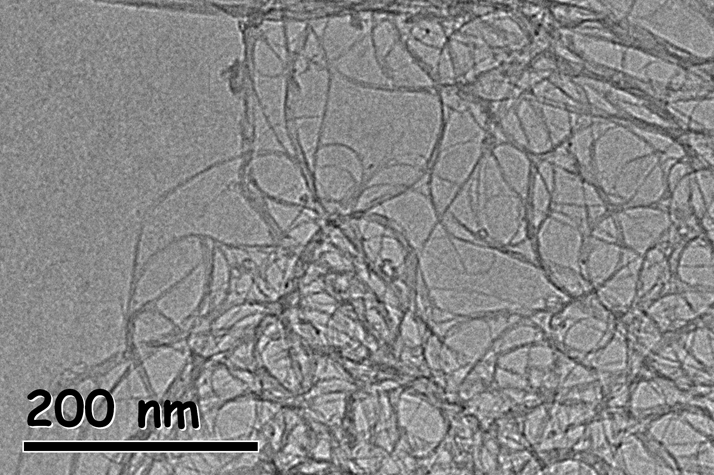


**Fig. S2.** TEM image of PPy-MWCNT





**Fig. S3.** Raman spectra of MWCNT and PPy-MWCNT

As seen in Fig S3, when MWCNT and PPy composite were formed, MWCNT was functionalized and I_D_/I_G_ value was increased as a result of increasing D band.


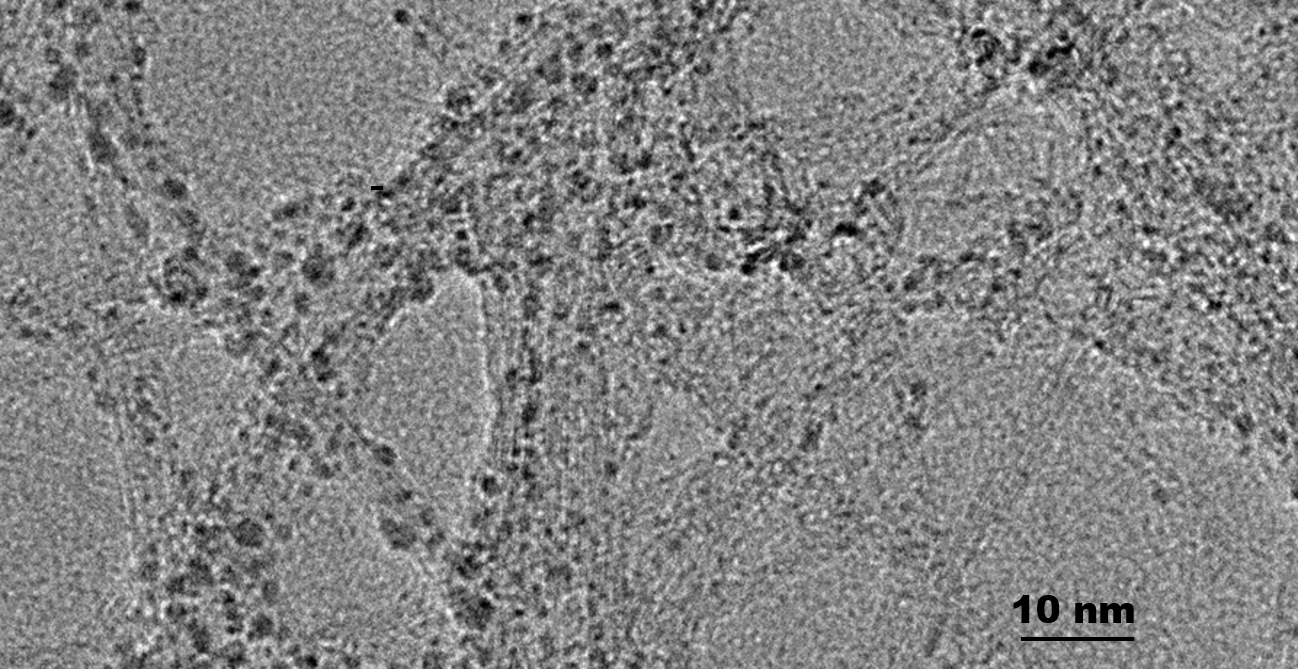


**Fig. S4.** TEM image of Pt@PPy-MWCNT

**
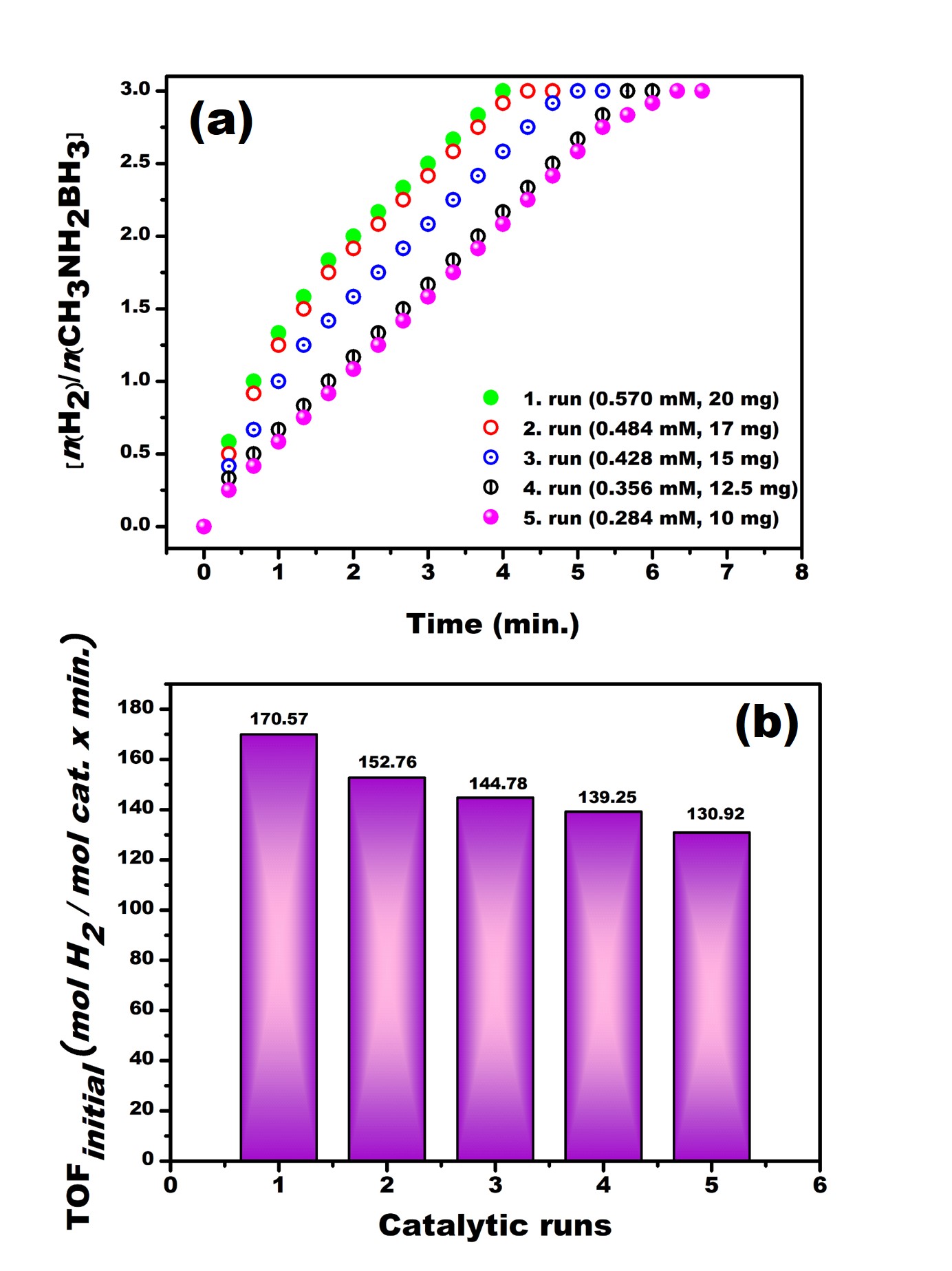
**

**Fig. S5.** (a) The performance of reusability and conversion % of Pt@PPy-MWCNT nanocatalyst for the hydrolytic dehydrogenation of MeAB, b) TOF for Pt@PPy-MWCNT catalyst versus the number of catalytic runs


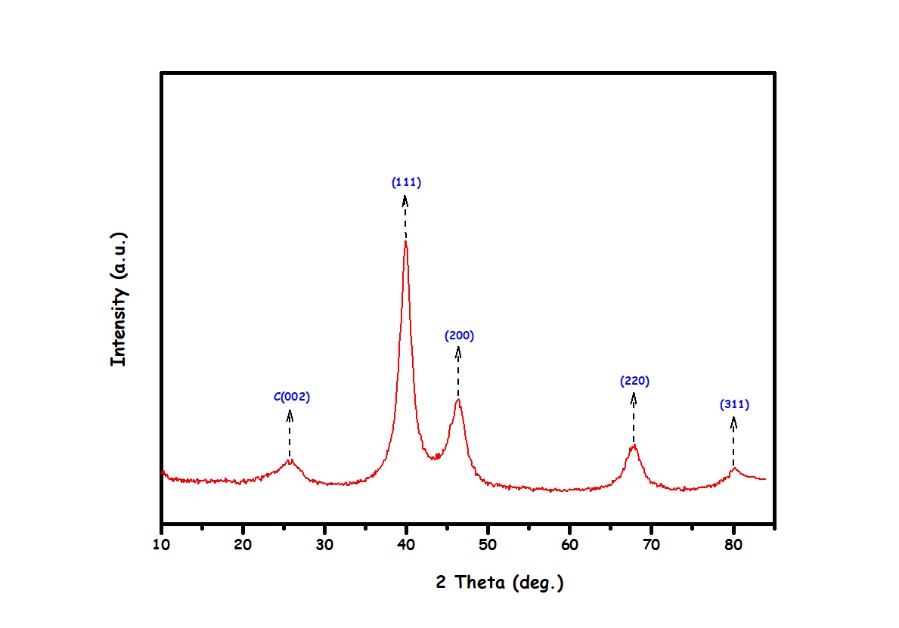


**Fig. S6.** XRD patterns of Pt@PPy-MWCNT (after reuse)


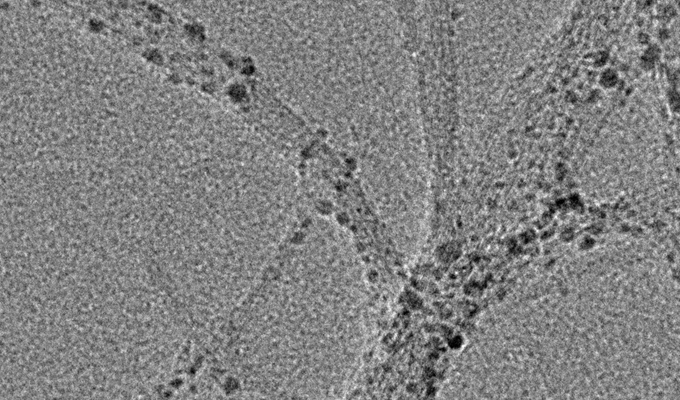


**Fig. S7.** TEM image of Pt@PPy-MWCNT (after reuse)
